# The responses of root morphology and phosphorus-mobilizing exudations in wheat to increasing shoot phosphorus concentration

**DOI:** 10.1093/aobpla/ply054

**Published:** 2018-09-20

**Authors:** Qi Shen, Zhihui Wen, Yan Dong, Haigang Li, Yuxin Miao, Jianbo Shen

**Affiliations:** 1 College of Resources and Environmental Science/Key Laboratory of Plant-Soil Interactions, Ministry of Education, China Agricultural University, Beijing, China; 2 College of Resources and Environment, Yunnan Agricultural University, Kunming, China; 3 College of Grassland, Resources and Environment, Inner Mongolia Agricultural University, Hohhot, China

**Keywords:** Rhizosphere, root, shoot phosphorus concentration, wheat

## Abstract

The adaptations of root growth and rhizosphere processes for soil phosphorus (P) acquisition have been investigated intensively in wheat (*Triticum aestivum*). However, only a few studies paid attention to these responses to shoot P status. This study aimed at investigating the responses of root morphology and P-mobilizing exudation to increasing shoot P concentration. A broad range of wheat shoot P concentrations (1.0–7.1 mg per g dry weight) was set up with 11 rates of P supply: 0–1200 mg P per kg soil. Root morphology and exudation parameters were measured after 37 days of plant growth. Shoot dry biomass reached a maximum when shoot P concentration was 4.63 mg per g dry weight. The maximum shoot P concentration for total root length, specific root length and the proportion of fine root (diameter ≤ 0.2 mm) length to total root length was 3 mg per g dry weight. Rhizosphere acidification was positively correlated with shoot P concentration when this was <5 mg per g dry weight. Shoot P concentration did not change acid phosphatase activity in the rhizosphere. Citrate concentration in the rhizosphere was suppressed by increasing shoot P concentration. In contrast, malate concentration in the rhizosphere showed a positive correlation with shoot P concentration. In conclusion, wheat root morphological and P-mobilizing exudation traits showed different behaviours with increasing P deficiency stress. Maintaining root biomass and length is the major strategy rather than root exudation for wheat to cope with extreme P deficiency.

## Introduction

Phosphorus (P) is one of the essential elements for plant growth and it is involved in many critical biochemical processes such as photosynthesis and respiration (Raghothama 1999). Soil P deficiency is a major constraint to crop yield in many parts of the world (Vance *et al.* 2003). In China, soil P in over 50 % of arable land is less than the agronomic optimum (Li *et al.* 2015b). Since phosphate rock may be exhausted in the next 50–100 years, P is a disappearing nutrient (Cordell *et al.* 2009; Gilbert 2009; Fixen and Johnston 2012; Johnston *et al.* 2014). Due to high P sorption of most soils, <20 % of applied fertilizer P may be uptaken by crops during the first growing season (Jones 1998; Zhang *et al.* 2008). Sorbed P in soil as legacy P has accumulated on many arable lands (Li *et al.* 2015b; Rowe *et al.* 2016). There is an urgent need to improve the biological potential of plants to efficiently utilize soil P.

Since phosphate is highly immobile in soil, diffusion is the main way that phosphate anions can reach the root surface (Lambers *et al.* 2008). Although the diffusion coefficient of phosphate in the soil is much less than that of other nutrients, the diffusion can be increased by increasing the phosphate concentration in the soil solution (Lambers *et al.* 2006). Moreover, dense root branching shortens diffusion distance of phosphate to the root surface and increases root interception (Lambers *et al.* 2006; Postma *et al.* 2014). In order to cope with P limitation, plants have evolved many morphological and physiological adaptations to enhance the roots’ P-uptake surface or mobilize unavailable soil P (Raghothama 1999; Vance *et al.* 2003).

Phosphorus-deficient plants often have a relatively greater root biomass and a larger root/shoot ratio than P-sufficient ones (Brouwer 1983; Lambers *et al.* 2006). The inhibition of primary root growth and proliferation of lateral root formation are enhanced by P deficiency, resulting in a shallow root system and increased total root length (Li *et al.* 2009; Péret *et al.* 2011; Niu *et al*. 2012). These traits help roots to exploit top soil efficiently where P is often rich (Niu *et al.* 2012). Plants tend to allocate more carbohydrates to roots which modified root/shoot ratio (Hermans *et al.* 2006). Wheat (*Triticum aestivum*) produces more fine roots in low P soil compared with high P soil (Yuan *et al.* 2016). It allows wheat to form a larger root surface area utilizing less carbon. Compared with maize (*Zea mays*), wheat has a similar level of response of root morphology to P deficiency in calcareous soil but a lower level of response in acid soil (Lyu *et al.* 2016).

Plants modify rhizosphere properties to increase soil P concentration in soil solution through root exudation including H^+^/OH^−^, carboxylates, phosphatase enzymes in P-limiting conditions (Hinsinger *et al.* 2003; Vance *et al.* 2003; Lambers *et al.* 2006). Rhizosphere acidification of P-deficient plants is well documented in many previous studies (Dinkelaker *et al.* 1989; Li *et al.* 2015a). The strong rhizosphere acidification of faba bean (*Vicia faba*) not only improves its own P uptake but also benefits P uptake of neighbouring maize plants in calcareous soil (Li *et al.* 2007). In contrast, faba bean has a greater OH^−^ release in acid soil (Li *et al.* 2015a). Carboxylates (e.g. citrate, malate) play an important role in mobilization of soil P (Lambers *et al.* 2008). Carboxylates exudation of roots is greatly induced by P deficiency (Hoffland *et al.* 1989a; Keerthisinghe *et al.* 1998). Acid phosphatases efficiently hydrolyse organic P compounds in soils (Tarafdar *et al.* 2001). The activity of these enzymes is much greater in rhizosphere of P-deficient plants compared with that in bulk soil (Gilbert *et al.* 1999; Wasaki *et al.* 2003).

Lyu *et al.* (2016) summarized root responses of major crops to P deficiency and grouped them into two categories: root morphology-based and physiology-based. Cereals often have a stronger root morphological responses than legumes to P deficiency, but legumes prefer to modify the root physiological process to mobilize soil P. This difference leads to complementary effects on P acquisition in intercropping systems (Li *et al.* 2018). Many factors can modify root responses to P deficiency, such as shoot P status and soil types (AđAlsteinsson *et al.* 1994; Shane *et al.* 2003; Erel *et al.* 2017). Shoot P status is the dominant factor to regulate these morphological and physiological adaptations to P deficiency (AđAlsteinsson *et al.* 1994; Shane *et al.* 2003; Li *et al.* 2008b). Shoot P concentration of wheat exerts a great role in regulating P influx rate and proportion of P transport to the shoot (AđAlsteinsson *et al.* 1994). Shoot P status regulates cluster-root growth and citrate exudation in white lupin (*Lupinus albus*) (Li *et al.* 2008b). However, only a few studies paid attention to trajectories of these responses with various shoot P status (Shane *et al.* 2003; Li *et al.* 2008b).

Wheat is one of the major food crops in the world, and consumes much more P fertilizer than rice and maize every year (Yuan *et al.* 2016). Therefore, improving P use efficiency of wheat is important for saving rock P reserves and reducing P loss to the environment. In this study, a broad range of shoot P status of wheat was created by applying different rates of P fertilizers. The objective was to investigate the responses of root morphology and P-mobilizing exudations in wheat to various shoot P status.

## Methods

### Experiment set-up

An experiment was conducted in a greenhouse with natural light at 28/16 °C (day/night, average temperature) and 45–55 % relative air humidity from September to October 2014. A calcareous silt loam soil was collected from Shangzhuang, Beijing, China (40°05′40″N, 116°12′32″E). The soil was air-dried and sieved to pass 2-mm screen, then mixed thoroughly. Soil properties were as follows: 8.40 (pH, soil:water ratio, 1:2.5), 11.5 g kg^−1^ (organic C); 0.51 g kg^−1^ (total N), 8.5 mg kg^−1^ (N_min_, NO_3_^−^ and NH_4_^+^), 0.69 g kg^−1^ (total P), 1.68 mg kg^−1^ (Olsen-P), 14.6 g kg^−1^ (total K), 82.4 mg kg^−1^ (NH_4_Ac exchangeable K). Each pot (volume of 1 L) was filled with 1 kg of air-dried soil. The nutrients were added to the soil as basal fertilizers at the following rates (mg kg^−1^): 1686.67 (Ca(NO_3_)_2_·4H_2_O), 335.10 (K_2_SO_4_), 125.67 (CaCl_2_), 43.34 (MgSO_4_·7H_2_O), 5.80 (EDTA-FeNa), 6.67 (MnSO_4_·4H_2_O), 10 (ZnSO_4_·7H_2_O), 2.0 (CuSO_4_·5H_2_O), 0.67 (H_3_BO_3_), 0.26 ((NH_4_)_6_Mo_7_O_24_·4H_2_O). Phosphorus was added to soil as KH_2_PO_4_ at a series of rates: 0, 2.5, 5, 10, 25, 50, 75, 150, 300, 600 and 1200 mg P per kg soil.

The wheat cultivar Yunmai 42 was selected for this study and the seeds were surface sterilized (30 min in a 10 % (v/v) H_2_O_2_ solution). They were germinated on the wet filter paper at 25 °C for 24 h in the dark. Six seeds were sown into each pot with 5 replicates per treatment. After 1 week, the seedlings were thinned to 3 plants per pot. During the whole experiment period, soil moisture in pots was kept at ~70 % field capacity by weighting.

### Sample collection and analysis

Plants were harvested after 37 days of growth in jointing stage, when visual growth differences among the P rate treatments were obvious **[see****Supporting Information—Fig. S1****]**. The wheat shoots were cut at soil surface. Roots adhered with soil were immerged into 0.2 mM CaCl_2_ solution as a trap solution, and they were shaken gently to collect the suspension solution of rhizosphere soil, which can be used to determine the pH, carboxylates and acid phosphatase in rhizosphere (Shen *et al.* 2003; Wang *et al.* 2007). During sampling, damage of fine roots and root hair should be avoided as much as possible. After sampling the rhizosphere exudation, all visible roots in each pot were then carefully picked out and stored in an ice cube box before they were transferred to the laboratory. In the laboratory, root samples were carefully cleaned using tap water and stored in a refrigerator before measurement of root morphological parameters. The bulk soil was also sampled. After air-drying, soil samples were ground to pass through a 2-mm sieve for analysis of bulk soil pH.

For carboxylates exudations determination, a 10-mL subsample of the rhizosphere extract was kept in a vial with addition of microbial inhibitor Micropur (Sicheres Trinkwasser, Rastatt, Germany) at 0.01 g L^−1^ and also three drops of concentrated phosphoric acid at −20 °C for high-performance liquid chromatography (HPLC) analysis. Carboxylates concentration in rhizosphere was measured using a reversed-phase HPLC (Shen *et al.* 2003). The chromatographic separation was carried out on a 250 × 4.6 mm reversed-phase column (Alltima C18, 5 µm; Alltech Associates, Inc., Deerfield, IL, USA). The mobile phase was 25 mmol L^−1^ KH_2_PO_4_ (pH 2.25) with a flow rate of 1 mL min^−1^ at 31 °C, and detection of the carboxylates was performed at 214 nm. For rhizosphere pH determination, the pH of the trap solution was measured, and adjusted to soil:water ratio of 1:2.5 based on the amount of rhizosphere soil (Li *et al.* 2010b). Acid phosphatase activity was determined using the spectrophotometric method based on the measuring of *p*-nitrophenol (PNP) absorbance at 405 nm (Alvey *et al.* 2001). The pH of bulk soil was measured after extraction in deionized water for 1 min at a soil:water ratio of 1:2.5.

Cleaned root samples were dispersed in water in a transparent array (30 × 20 × 3 cm) and scanned at a resolution of 400 dpi (Epson Expression 1600, Seiko Epson, Nagano, Japan). The root traits were determined by analysis of images using WinRHIZO software (Regent Instrument, Quebec, Canada). The shoots and roots were washed with the deionized water and then oven-dried at 70 °C for 3 days. After being weighed, plant materials were ground to powder for nutrient analysis. To determine the plant total P concentration, dried samples were milled and subsequently digested with concentrated H_2_SO_4_ and H_2_O_2_ using the molybdate-blue colorimetric method (Murphy and Riley 1962). Total P was measured with the vanado-molybdate method (Westerman 1990) from the P concentration in the digest.

### Data analysis

Analysis of variance (ANOVA) was conducted using the one-way ANOVA model in the SAS statistical software (SAS 8.1, USA). Significant differences among means were determined by LSD at the *P* ≤ 0.05 probability level. Relationships between root/shoot ratio, shoot P concentration, rhizosphere physiological traits and root morphological traits were plotted using the SigmaPlot statistical software (SigmaPlot 10.0, USA).

The linear-plateau model was used for analysis of the relationship between shoot dry biomass, root dry biomass, root/shoot ratio, total root length and shoot P concentration. The unimodal model was established for analysis of relationship between specific root length, proportion of wheat root length in different diameter classes, rhizosphere change value and shoot P concentration. When it reached the maximum then the data witnessed a sharp decrease trend, so we chose the unimodal model. Linear model was used for analysis of the relationship between citrate, malate and shoot P concentration when there is no critical point.

## Results

### Plant growth and P uptake as affected by P rates

Shoot biomass increased significantly when the P addition rate was >10 mg kg^−1^, and eventually reached 1.68 g dry weight per pot at a P addition rate of 1200 mg kg^−1^, which was 5.72 times higher than that in the treatment without P addition (Table 1). Shoot P concentration also increased from 1.0 to 7.1 mg P per g dry weight with increasing P addition rates. The total root length and specific root length increased with increasing P supply when P addition rate was <25 mg kg^−1^. The specific root length declined slightly when P addition rates increased from 75 to 1200 mg kg^−1^. Roots were divided into three categories based on root diameter including fine roots (diameter < 0.2 mm), medium-sized roots (diameter between 0.2 and 0.4 mm) and thick roots (diameter > 0.4 mm). The proportion of fine roots length (diameter < 0.2 mm) to total root length reached a maximum (84.6 %) at P addition rate of 50 mg kg^−1^. Both lower and higher P addition rates reduced the proportion of fine root length to total root length (PFR), which was the lowest (69.5 %) in the treatment without P addition. The proportion of medium-sized root length to total root length (PMR), ranged from 13.7 to 24.3 %, was the highest in the treatment without P addition. The proportion of thick root to total root length (PTR) was <10 % in all the treatments.

**Table 1.** Biomass, P concentration, total and specific root length and proportion of root length with different diameters to total root length of wheat grown with different P supplies. Each value is the mean (±SE) of five replicates. Different letters in a given column denote significant differences among P rates (*P* ≤ 0.05).

P rate (mg kg^−1^)	Shoot biomass (g per pot)	Root biomass (g per pot)	Shoot P concentration (mg g^−1^)	Total root length (m per pot)	Specific root length (m g^−1^)	Proportion of root length in different diameters to total root length (%)		
						<0.2 mm	0.2–0.4 mm	>0.4 mm
0	0.25 ± 0.01f	0.15 ± 0.00f	0.98 ± 0.03h	26.3 ± 1.2d	173 ± 6f	69.5 ± 0.7g	24.3 ± 0.6a	6.2 ± 0.3bc
2.5	0.30 ± 0.01f	0.18 ± 0.01ef	0.97 ± 0.03h	32.2 ± 1.5dc	180 ± 3f	70.9 ± 1.0gf	23.6 ± 0.8ab	5.5 ± 0.2cd
5	0.28 ± 0.02f	0.18 ± 0.01ef	1.09 ± 0.06h	37.5 ± 2.7dc	210 ± 8e	73.8 ± 0.9efg	21.3 ± 0.6bc	4.9 ± 0.4de
10	0.32 ± 0.02f	0.19 ± 0.01e	1.23 ± 0.03h	43.3 ± 4.3c	224 ± 9de	76.4 ± 1.3cd	19.4 ± 1.1cd	4.2 ± 0.3efg
25	0.68 ± 0.04e	0.26 ± 0.02d	2.35 ± 0.06g	78.4 ± 6.1ab	295 ± 8a	81.3 ± 1.1b	15.3 ± 1.1efg	3.4 ± 0.2g
50	1.06 ± 0.05d	0.28 ± 0.01d	3.18 ± 0.05f	80.6 ± 8.0ab	289 ± 18ab	84.6 ± 0.9a	11.9 ± 0.6h	3.5 ± 0.3fg
75	1.25 ± 0.03c	0.32 ± 0.01ab	3.68 ± 0.09e	84.0 ± 4.7a	265 ± 15abc	81.8 ± 0.8ab	13.7 ± 0.8gh	4.5 ± 0.2def
150	1.49 ± 0.03b	0.29 ± 0.01bcd	4.71 ± 0.13d	75.7 ± 5.2ab	261 ± 8bc	78.9 ± 1.6bc	15.0 ± 1.1fg	6.1 ± 0.5c
300	1.44 ± 0.05b	0.28 ± 0.02cd	5.08 ± 0.19c	69.6 ± 7.0b	244 ± 10cd	76.1 ± 1.1cd	16.6 ± 0.7ef	7.3 ± 0.4ab
600	1.45 ± 0.05b	0.31 ± 0.01abc	5.83 ± 0.15b	79.3 ± 2.4ab	252 ± 8cd	75.1 ± 0.9de	17.5 ± 0.7de	7.4 ± 0.2a
1200	1.68 ± 0.12a	0.34 ± 0.01a	7.12 ± 0.05a	82.9 ± 5.3ab	245 ± 15cd	72.5 ± 1.8efg	19.4 ± 1.1cd	8.2 ± 0.8a

### Relationship between plant growth and shoot P concentration

**Figure 1.**
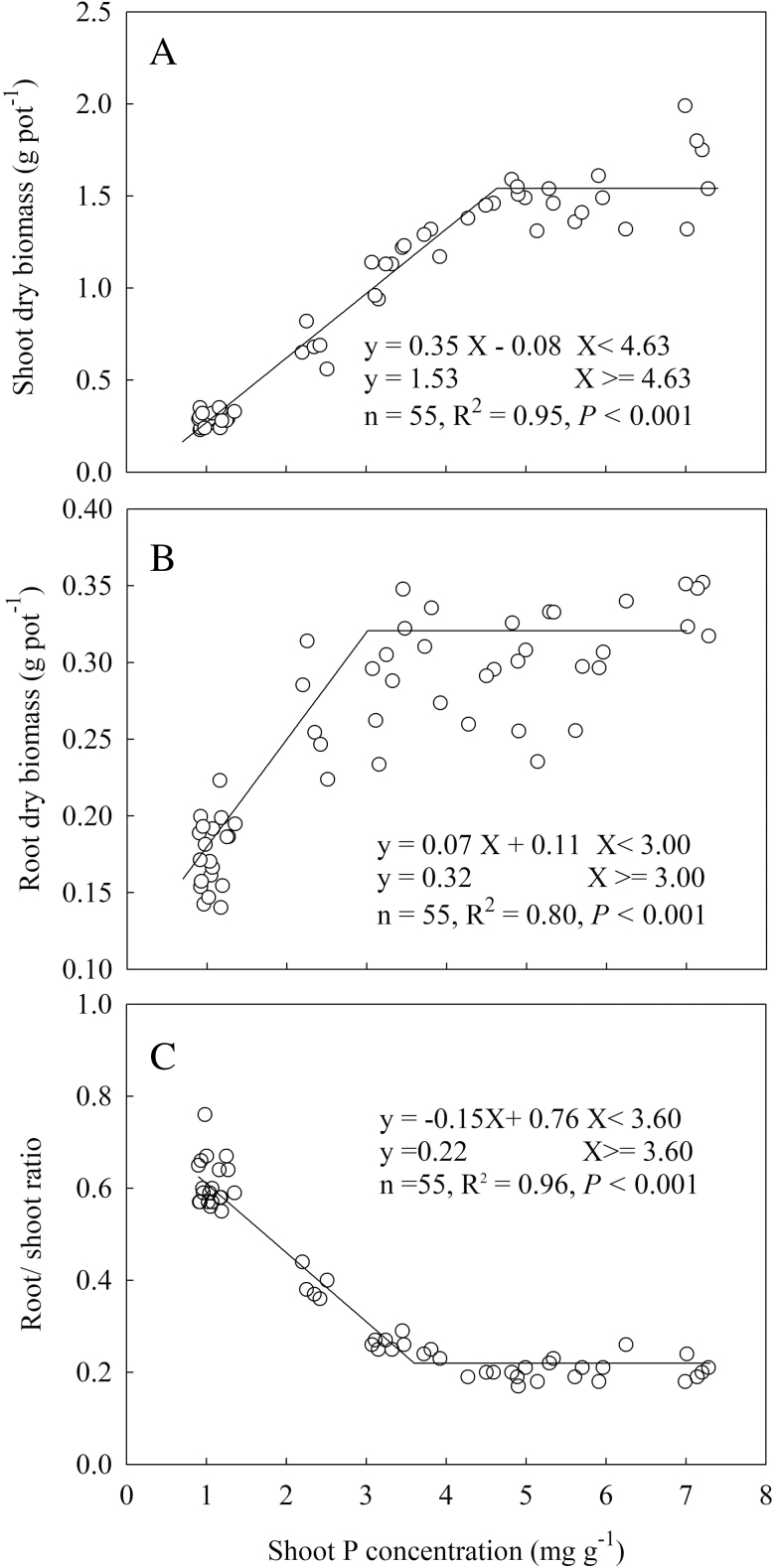
Shoot biomass (A), root biomass (B) and root/shoot ratio (C) in response to shoot P concentration. Data point represents individual replicate.

The relationship between shoot biomass and shoot P concentration was fitted well by a linear-plateau model (*R*^2^ = 0.96, *P* < 0.001) (Fig. 1A). Shoot biomass reached a plateau when shoot P concentration was 4.63 mg P per g and above, below which shoot biomass continuously decreased with decreasing shoot P concentration. A linear-plateau model also fitted the relationship between root biomass and shoot P concentration (*R*^2^ = 0.80, *P* < 0.001) (Fig. 1B). With root biomass increasing with shoot P concentration, and when shoot P concentration surpassed 3.00 mg P per g, root growth levelled off, which was earlier than that of shoot biomass. The root/shoot ratio declined substantially with increasing shoot P concentration, reaching a plateau at 3.60 mg P per g (Fig. 1C).

**Figure 2.**
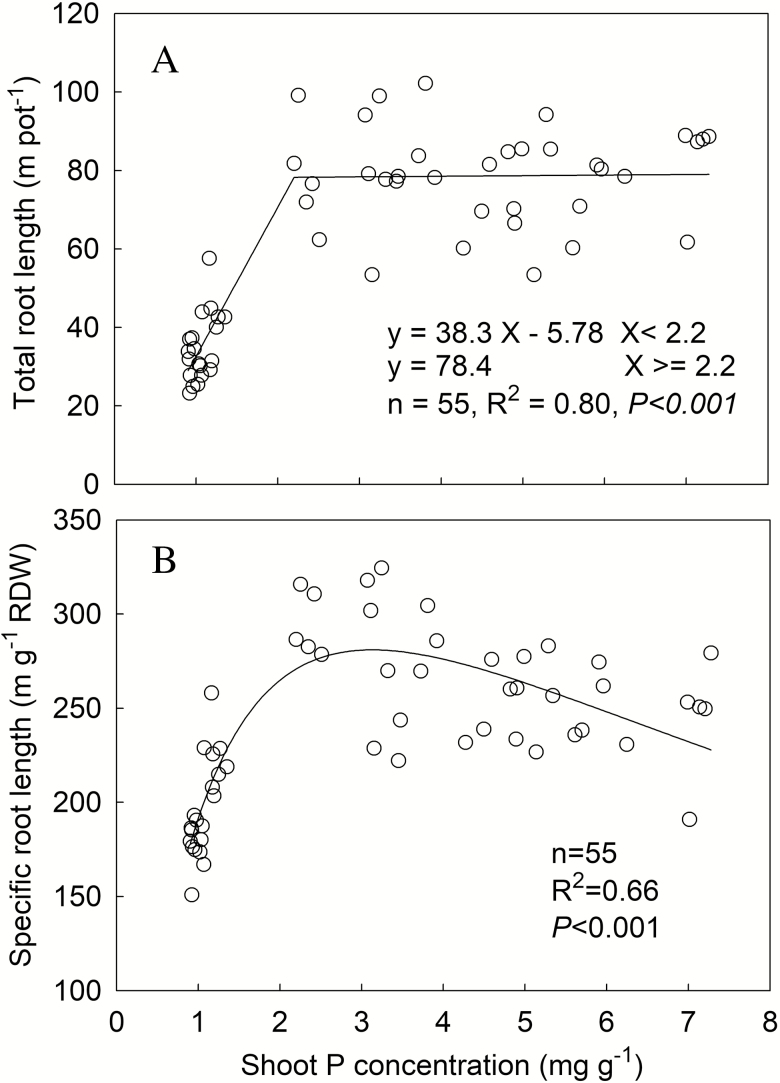
Total root length (A) and specific root length (B) in response to shoot P concentration. Data point represents individual replicate. RDW, root dry weight.

The relationship between total root length and shoot P concentration was also fitted well by a linear-plateau model (*R*^2^ = 0.80, *P* < 0.001) (Fig. 2A). The total root length was positively correlated with shoot P concentration when it was <2.2 mg P per g, above which total root length did not significantly change and was maintained at 78.9 m per pot with increasing shoot P concentration. The change of specific root length with increasing shoot P concentration showed a unimodal pattern (*R*^2^ = 0.66, *P* < 0.001). The specific root length increased from 177 to a peak of 281 m per g root dry weight at 3 mg g^−1^ for shoot P concentration and then continuously decreased to 228 m per g root dry weight (Fig. 2B).

**Figure 3.**
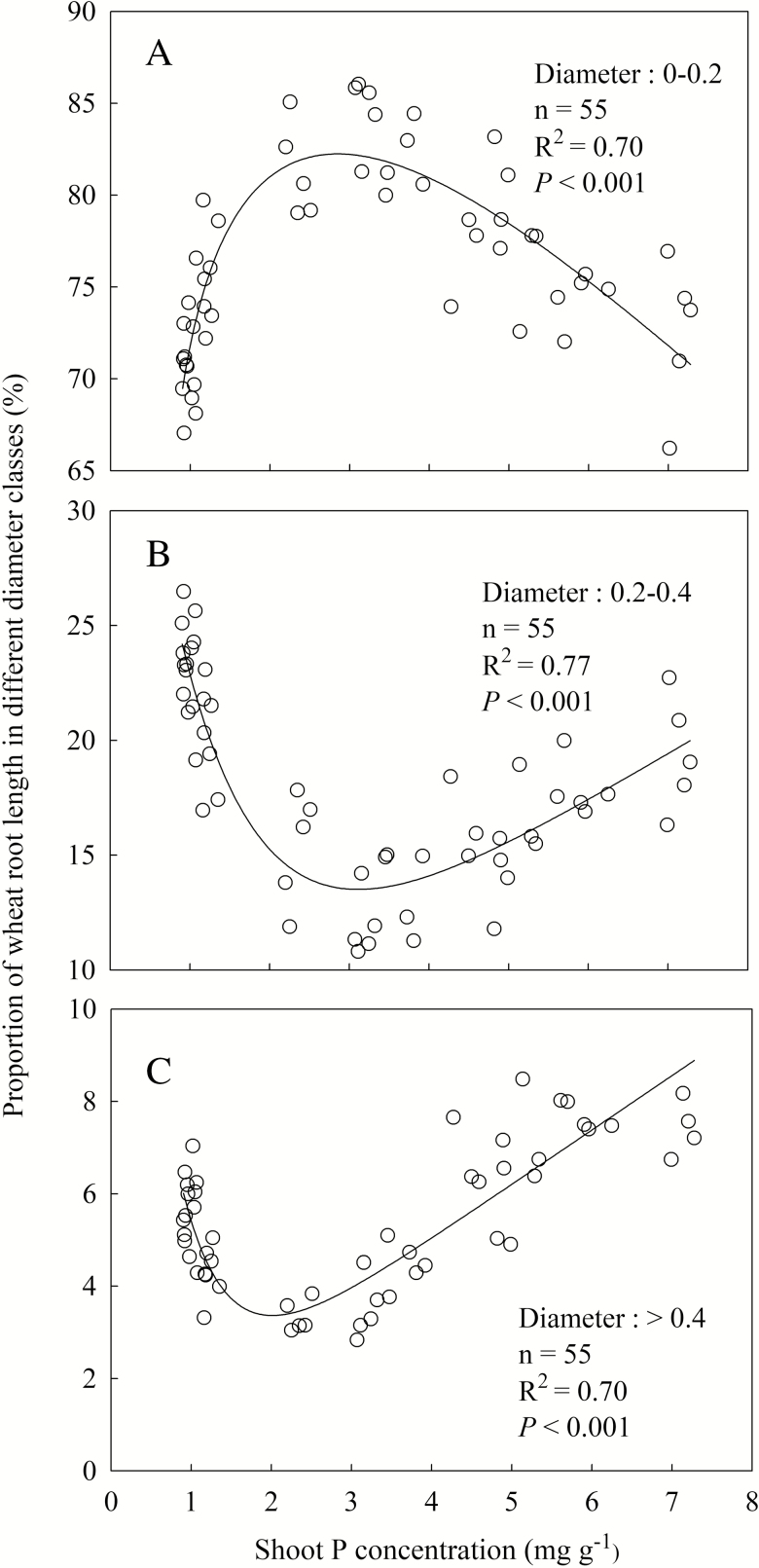
The proportion of root length with different diameter classes to total root length in response to increasing shoot P concentration. (A) Fine root in root diameter class: 0–0.2 mm; (B) medium-sized root in root diameter class: 0.2–0.4 mm; (C) thick root in root diameter class: >0.4 mm. Data point represents individual replicate.

The PFR showed a unimodal pattern with increasing shoot P concentration (*R*^2^ = 0.70, *P* < 0.001), which was similar to that of specific root length (Fig. 3A). The maximum shoot P concentration was 3 mg g^−1^, below which PFR increased with decreasing shoot P concentration, and above which, PFR decreased. The PMR and PTR showed an opposite pattern to PFR with increasing shoot P concentration (Fig. 3B and C). The PMR and PTR reached a minimum when shoot P concentration was 3 and 2 mg g^−1^, respectively, above which the proportions continuously increased with increasing shoot P concentration.

### Relationship between rhizosphere processes and P addition rates

The bulk soil pH at harvest was ~8.2 in the treatments ranging from P0 to P600, except the treatment with 1200 mg kg^−1^ showing a slight reduction by 0.28 unit (Table 2). The rhizosphere pH was not significantly different from the bulk soil pH when the amount of P addition was below 10 mg kg^−1^. A significant acidification of the rhizosphere was observed when P addition rates were >25 mg kg^−1^. The pH decreased to the minimum level when P addition rate was above 150 mg kg^−1^. The maximum acid phosphatase activity in the rhizosphere was observed when P addition rate was 25 mg kg^−1^, which was 234 µg PNP per h per g soil on average. In contrast, the minimum value was 91.5 µg PNP per h per g soil when P addition rate was 75 mg kg^−1^. The citrate and malate concentration in the rhizosphere ranged from 107 to 244 and 0 to 313 nmol per g soil, respectively. The highest concentration was observed when P addition rate was 1200 mg P per kg soil for malate and was 10 mg P per kg soil for citrate.

**Table 2.** Soil pH, acid phosphatase activity, and citrate and malate concentration in the rhizosphere of wheat grown with different P supplies. Each value is the mean (±SE) of five replicates. Different letters in a given column denote significant differences between P rates (*P* ≤ 0.05).

P rate (mg P per kg soil)	Rhizosphere soil pH	Bulk soil pH	Rhizosphere soil pH change	Acid phosphatase activity (μg PNP per h per g soil)	Citrate (nmol per g soil)	Malate (nmol per g soil)
0	8.00 ± 0.05a	8.19 ± 0.01ab	0.18 ± 0.05d	117 ± 3cd	237 ± 29bcd	0 ± 0d
2.5	8.02 ± 0.02a	8.20 ± 0.00a	0.18 ± 0.02d	162 ± 18bc	244 ± 15abc	0 ± 0d
5	7.97 ± 0..05a	8.19 ± 0.01ab	0.22 ± 0.05d	203 ± 31ab	260 ± 34ab	44 ± 44d
10	7.91 ± 0.04a	8.22 ± 0.01a	0.31 ± 0.05d	175 ± 18abc	295 ± 29a	31 ± 31d
25	7.62 ± 0.05b	8.20 ± 0.01a	0.59 ± 0.06c	234 ± 31a	194 ± 16cde	143 ± 43c
50	7.56 ± 0.04b	8.19 ± 0.01ab	0.63 ± 0.04c	190 ± 51ab	195 ± 22cde	185 ± 16bc
75	7.42 ± 0.04c	8.22 ± 0.01a	0.80 ± 0.05b	92 ± 6d	167 ± 16ef	192 ± 19bc
150	7.36 ± 0.03cd	8.20 ± 0.00a	0.84 ± 0.03ab	122 ± 16cd	168 ± 25def	259 ± 40ab
300	7.33 ± 0.06cd	8.15 ± 0.02b	0.82 ± 0.06ab	153 ± 13bcd	138 ± 17ef	214 ± 23bc
600	7.27 ± 0.05d	8.21 ± 0.04a	0.95 ± 0.06a	146 ± 16bcd	121 ± 15f	261 ± 39ab
1200	7.28 ± 0.03d	7.91 ± 0.04c	0.63 ± 0.02c	166 ± 15abc	107 ± 16f	313 ± 28a

### Relationship between rhizosphere processes and shoot P concentration

**Figure 4.**
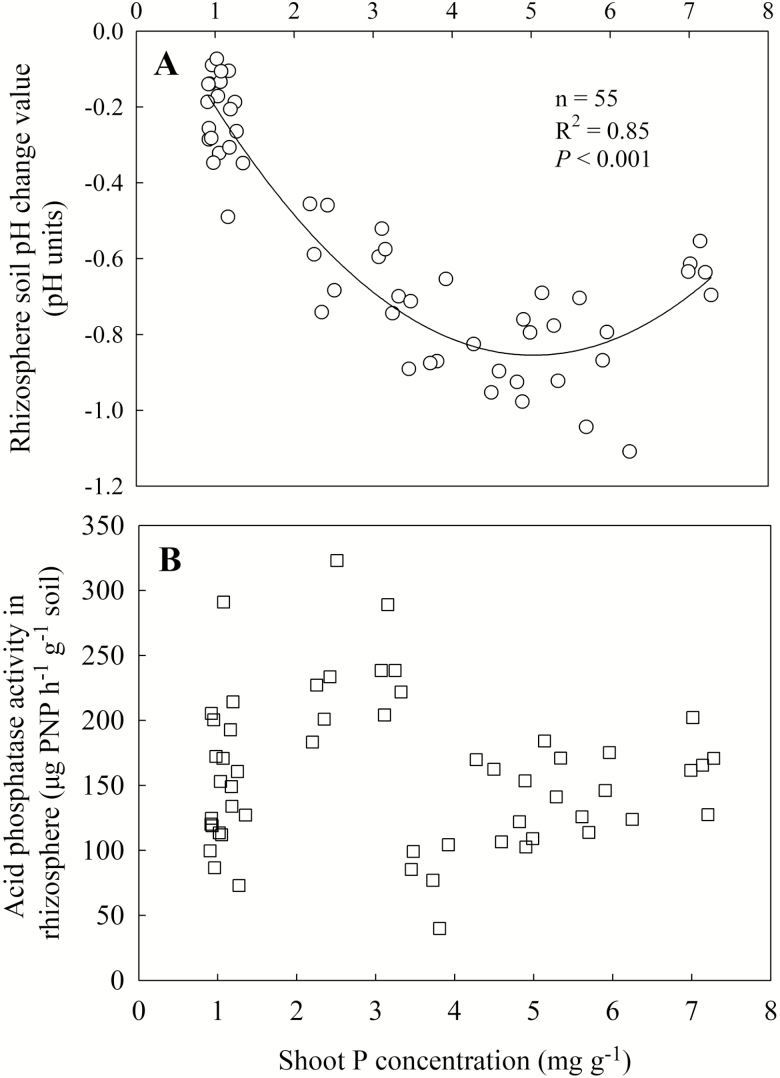
Rhizosphere soil pH change (bulk soil pH minus rhizosphere soil pH) (A) and acid phosphatase activity in rhizosphere (B) in response to shoot P concentration. Data point represents individual replicate.

There was a significant acidification in the rhizosphere of wheat at a high shoot P concentration till 5.0 mg g^−1^, at which rhizosphere pH decreased by 0.8 unit (Fig. 4A). With shoot P concentration increased to 7.0 mg g^−1^, rhizosphere acidification was slightly reduced to 0.7 unit.

**Figure 5.**
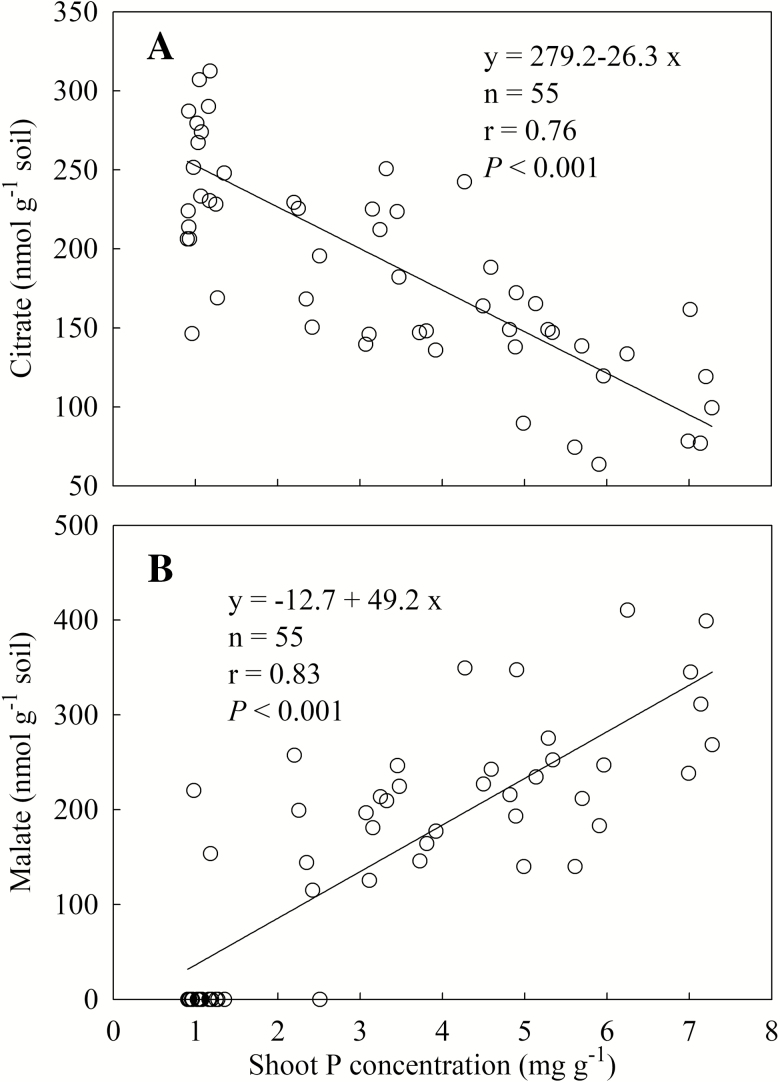
Concentration of citrate (A) and malate (B) in the rhizosphere in response to shoot P concentration. Data point represents individual replicate.

There was a large variation in acid phosphatase activity in the rhizosphere (from 49 to 310 µg PNP per h per g soil), especially, when shoot P concentration was around 1.0 mg g^−1^ (Fig. 4B). No correlation was observed between acid phosphatase activity and shoot P concentration. There was a negative correlation between citrate concentration in the rhizosphere and shoot P concentration (*R*^2^ = 0.58, *P* < 0.001) (Fig. 5A). In contrast, the malate concentration in the rhizosphere showed a positive correlation with shoot P concentration (Fig. 5B).

## Discussion

### Plant growth and P uptake

There is a critical shoot P concentration for plant growth, and it depends on growth stages (Jones 1983; Sandaña and Pinochet 2014; Bélanger *et al.* 2015). The range of critical shoot P concentration for wheat growth is quite wide, being 1.3–2.8 mg g^−1^ for young whole shoots and 2.7–3.6 mg g^−1^ for recently matured leaves (Rashid *et al.* 2005). A high critical shoot P concentration is observed by Reuter *et al.* (1997) that is 3.8–4.5 mg g^−1^ for young whole shoots and 4.4–4.7 % for recently matured leaf blades. Differences of genotypes and achieved yields are presumed to contribute to this discrepancy. In this study, a broad range of shoot P concentration was created by adding a series of P amounts into soil, and the critical shoot P concentration was 4.63 mg g^−1^ for shoot growth and was only 3.00 mg g^−1^ for root growth. This indicated that shoot growth suffered P deficiency stress earlier than root at early growth stage. Maintaining root growth facilitates P-deficient plants to absorb soil P, which is an effective adaptation strategy to copy with P deficiency stress. This is in contrast to the root response of white lupin (Li *et al.* 2010a), which prefers to enhance exudation of carboxylates and acid phosphatases to mobilize soil P than produce more roots to increase soil P adsorption surface under P-deficient conditions (Lyu *et al.* 2016). Moreover, the cluster-root induced by P deficiency concentrates P-solubilizing compounds released by rootlets in a limited area, and then magnifies P-mobilizing capacity of roots (Hocking *et al.* 1998; Watt and Evans 1999).

### Root morphological traits

In order to enhance P acquisition under limited P supply situations, plant often modifies root morphological traits to increase the ability of root to absorb P from soil (Schjørring and Nielsen 1987). Larger root system provides greater adsorption surface for soil nutrients, which is particularly important for soil P as a less mobile ion (Singh Gahoonia and Nielsen 2004). It was confirmed by Brück *et al.* (1992) when comparing plant P uptake between ‘rootless’ maize mutant and wild type. More biomass is allocated to roots when plant is suffering P deficiency (Lambers *et al.* 2006). The P-deficient wheat had a larger root/shoot ratio than P-sufficient plants with >50 mg P per kg addition in this study (Table 1). Root/shoot ratio was enhanced by increasing P stress, indicating that biomass allocation between shoots and roots is systemically regulated by shoot P status. Reduction of root biomass caused by P deficiency (shoot P concentration is below 3.00 mg g^−1^) did not result in reduction of total root length until shoot P concentration decreased to 2.2 mg g^−1^. Wheat had the highest specific root length within this range of shoot P concentration because of fine roots proliferation, indicating that wheat produces more root length with less root biomass. It is consistent with the results of Yuan *et al.* (2016) who found that P deficiency enhanced root length density and reduced root biomass at the same time, and intensity of these responses was dependent on soil types (Yuan *et al.* 2016). With increasing P deficiency (<2.2 mg g^−1^), root length was finally reduced. This is opposite to the previous research that sugar beet has a greater root length in low-P plots than high-P plots (Steingrobe 2001). In contrast, Ma and Rengel (2008) observed a similar pattern as this study. This response should be P deficiency intensity dependent: light P deficiency only reduces root biomass but not root length and extreme P deficiency reduces both. A smaller root diameter results in larger root adsorption surface per unit biomass (Atkinson 1990). The P deficiency enhances specific root length and fine root production in some cases (Christie and Moorby 1975; Schroeder and Janos 2005). However, this response was observed only when shoot P concentration was >3 mg g^−1^ in this study, below which the fine root production was suppressed by P deficiency. A root proliferation was induced by P deficiency at early stage, and finally disappeared with increasing stress intensity caused by plant growth (Marschner 2011). We possibly missed early response of wheat after a long growth period (37 days after planting) in low P soil. Decrease of fine root growth was possibly due to reduction of the numbers of lateral roots and lateral root primordia caused by extreme P deficiency (Li *et al*. 2012). This response is co-regulated by DNA replication, transcription, protein synthesis and degradation and cell growth. Although fine roots are more efficient than thick ones, carbon cost of fine roots may be much greater because of more frequent turnover (Persson 1983). Hence, it is speculated that the fine roots of wheat were more inhibited at extremely low P condition compared with thick roots (diameter > 0.2 mm).

### The release of root exudates

To increase P mobilization, root morphological changes always follow with physiological changes, such as root exudation (Vance *et al.* 2003; Lambers *et al.* 2006). Soil pH plays a prominent role in chemical equilibrium of soil P that determines soil P bioavailability (Hinsinger 2001; Hinsinger *et al.* 2003). Soil acidification enhances dissolution of Ca phosphates to increase soil P bioavailability in calcareous soil (Hinsinger 2001; Li *et al.* 2015a). Many plant species reduce medium pH in low P condition, such as white lupin, tomato (*Lycopersicon esculentum* L.) and chickpea (*Cicer arietinum* L.) (Neumann and Römheld 1999). However, rhizosphere acidification of P-deficient wheat was weak in the previous studies (Neumann and Römheld 1999; Li *et al.* 2008a). No significant acidification was observed for extreme P-deficient wheat as well in this study. On the contrary, rhizosphere acidification strengthened with the increasing shoot P concentration till 5.0 mg g^−1^ (Fig. 4A), which was possibly due to excessive uptake of cations by root than anions (Hinsinger *et al.* 2003). In this study, P was added into soil as KH_2_PO_4_; thus, K as an accompany ion was inevitably added. Wheat absorbed much more K per unit root length with high P additions than low P additions **[see****Supporting Information—Fig. S2****]**, which led to rhizosphere acidification of wheat with sufficient P supply, as shown in the previous study (Wen *et al.* 2017). The P-deficient wheat did not depend on rhizosphere acidification to mobilize soil P, which was consistent with the previous study (Li *et al.* 2008a). Phosphatase activity in the rhizosphere has a tight positive correlation with the depletion of soil organic P (Tarafdar and Jungk 1987). The activity of acid phosphatase is often high in the rhizosphere of P-deficient plants (Gilbert *et al.* 1999; Wasaki *et al.* 2003; Ciereszko *et al.* 2011), which was up to eight times greater than bulk soil (Tarafdar and Jungk 1987). The relative expression levels of purple acid phosphatase genes (*PAP15* and *PAP16*) of wheat were down-regulated with increasing soil P supply at flowering stage (Teng *et al.* 2013). However, phosphatase activity in the rhizosphere of maize had a positive linear correlation with shoot P concentration (Wen *et al.* 2017). This response may facilitate recapture of some organic P lost from roots into the rhizosphere. In this study, we failed to find correlation between acid phosphatase activity and P addition rates or shoot P concentration. In unsterilized condition, acid phosphatase in the rhizosphere is partly from soil microorganisms (Tarafdar *et al.* 1992). The change of acid phosphatase secretion of roots induced by variable shoot P concentration may be covered by secretion of soil microorganisms in this study.

Phosphorus-deficient plants exude carboxylates into rhizosphere to mobilize soil P through complexing metal cations-bound phosphate and displacing phosphate from soil mineral surface by ligand exchange (Hoffland *et al.* 1989b; Gerke *et al.* 2000; Jones *et al.* 2003). Predominant carboxylate composition is malate and citrate in the rhizosphere of wheat, and malate comprised over 85 % of total carboxylate (Pearse *et al.* 2006). However, carboxylate is not observed in the rhizosphere of wheat in other studies (Li *et al.* 2008a; Rose *et al.* 2010). We found malate and citrate in the rhizosphere of wheat, which is consistent with the results of Pearse *et al.* (2006). The carboxylate exudation of wheat is shoot P status-dependent. Increasing shoot P concentration diminished citrate accumulation, but induced malate accumulation synchronously. It is opposite to the results of Pearse *et al.* (2006) that wheat produced similar amounts of carboxylates between P-deficient and -sufficient plants (Pearse *et al.* 2006). Maize enhances carboxylates exudation with increasing shoot P concentration in some studies (Liu *et al.* 2004; Li *et al.* 2010b, Wen *et al.* 2017). This response may not be an adaptation to P deficiency but a by-product of high root activity of plants. Thus, only citrate exudation of wheat was regarded as an adaptive response to P deficiency in this study.

It should be noted that damage of root and root hair during the sampling could change root exudation, and then change carboxylate concentration in rhizosphere no matter how root sampling is carefully processed. This may cause overestimation of carboxylate concentration in rhizosphere in this study.

In summary, plant growth was suppressed by P deficiency in the following order: shoot biomass > root/shoot ratio > root biomass > total root length. It indicated that shoot growth was more sensitive to shoot P status than roots. As a root morphology-based crop, wheat did not have consistent root physiological traits to cope with P deficiency. Shoot P concentration as a dominant factor regulated these adaptations of soil P acquisition for wheat.

## Conclusions

Root morphological and physiological traits of wheat showed different behaviours with increasing P deficiency. Phosphorus-deficient wheat preferred to maintain root growth than shoot by allocating more carbon to root, and root length by producing more fine roots even root biomass decreased. Extreme P deficiency (shoot P concentration < 3 mg g^−1^) reduced more fine roots production than thick roots. Wheat did not increase rhizosphere acidification and acid phosphatase secretion in low P soil. Citrate not malate exudation of roots as an adaptive response was enhanced by P deficiency. It is concluded that maintaining root biomass and length is the major strategy for wheat to cope with extreme P deficiency rather than root exudation.

## Contributions by the Authors

Q.S., H.L. and J.S. designed the experiments; Q.S. and Z.W. performed the experiment and analysed the data; the authors interpreted the data together; Q.S. wrote the first draft of the manuscript; H.L. coordinated the research and supported all the technical support. All authors participated in writing the manuscript.

## Conflict of Interest

None declared.

## Supplementary Material

Supplementary Materials

## Supporting Information

The following additional information is available in the online version of this article—

**Figure S1.** The response of wheat shoots to different P fertilization rates (in mg P per kg soil) at the harvest time (37 days after planting).

**Figure S2.** The rhizosphere soil pH change (bulk soil pH minus rhizosphere soil pH) with K acquisition efficiency (total K uptake divide total root length). Each symbol represents the mean of five replicates (±SD). The numbers above each symbol indicate P added rates (mg kg^−1^).
