# Supplementary material for: The responses of root morphology and phosphorus-mobilizing exudations in wheat to increasing shoot phosphorus concentration
Source: AoB Plants. 2018 Sep 20;10(5):ply054. doi: 10.1093/aobpla/ply054 (PMC6185719; doi:10.1093/aobpla/ply054)
Supplement: Supplementary Materials [file ply054_suppl_supplementary_materials.pdf]

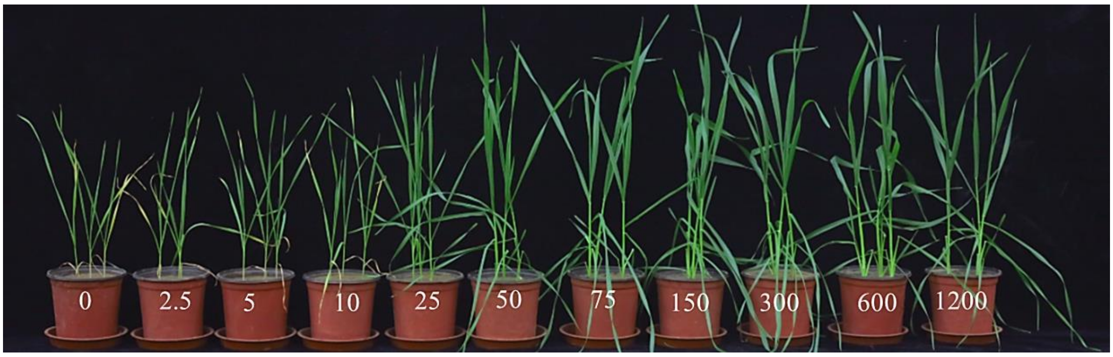

Fig. S1 The response of wheat shoots to different P fertilization rates (in mg P kg^{-1} soil) at the harvest time (37 days after planting).

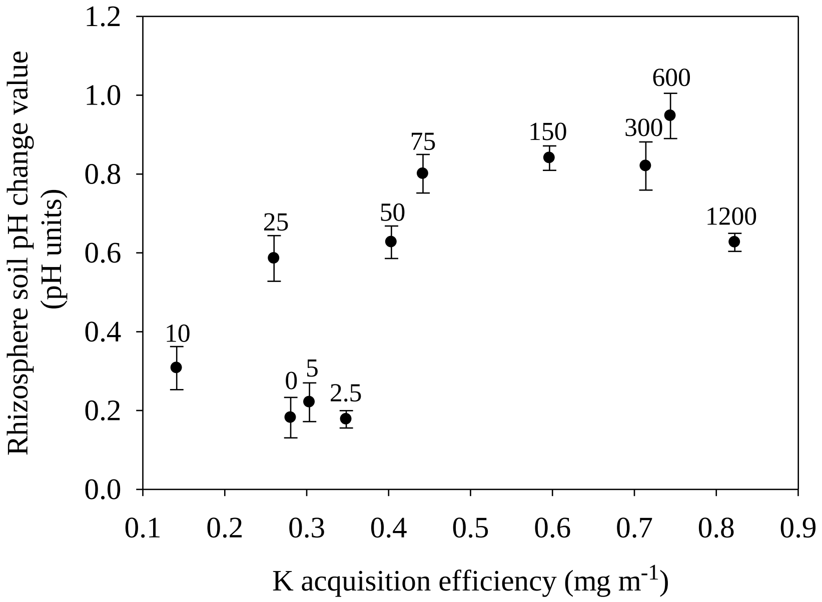

Fig. S2 The rhizosphere soil pH change (bulk soil pH minus rhizosphere soil pH) with K acquisition efficiency (total K uptake divide total root length). Each symbol represents the mean of five replicates (±SD). The numbers above each symbol indicate P added rates (mg kg^{-1}).
